# Supplementary material for: Making genomic surveillance deliver: A lineage classification and nomenclature system to inform rabies elimination
Source: PLoS Pathog. 2022 May 2;18(5):e1010023. doi: 10.1371/journal.ppat.1010023 (PMC9162366; doi:10.1371/journal.ppat.1010023)
Supplement: S2 Table — Details of the 14 designated lineages from lineage designation run exclusively on whole genome (10000nt) rabies virus sequences from Tanzania, obtained from RABV-GLUE. Includes all places sequences assigned each lineage have been seen in, the first and most recent collection years of those sequences, the maximum and mean patristic distance of all sequences in each lineage and the lineages descended from it, and the number of sequences assigned to each lineage. Where place = NA, no information is publicly available for sequence locations. Mara region contains the Serengeti District. (DOCX) [file ppat.1010023.s002.docx]

| ***lineage*** | ***place*** | ***year_first*** | ***year_last*** | ***max_patristic_dist*** | ***mean_patristic_dist*** | ***n_seqs*** |
| --- | --- | --- | --- | --- | --- | --- |
| ***Cosmopolitan AF1b_A1*** | *Morogoro region, Mara region* | *2009* | *2017* | *0.07* | *0.02* | *7* |
| ***Cosmopolitan AF1b_A1.1*** | *Arusha Region, Mtwara region, Pemba island, Mara region* | *2003* | *2011* | *0.07* | *0.02* | *4* |
| ***Cosmopolitan AF1b_A1.1.1*** | *Pwani region* | *2010* | *2018* | *0.07* | *0.02* | *2* |
| ***Cosmopolitan AF1b_A1.1.2*** | *Arusha Region, Dar es Salaam, Iringa region, Kusini Unguja (Zanzibar), Lindi region, Morogoro region, Pemba island, Mara region* | *1996* | *2017* | *0.07* | *0.02* | *37* |
| ***Cosmopolitan AF1b_B1*** | *Mara region* | *2004* | *2012* | *0.07* | *0.02* | *11* |
| ***Cosmopolitan AF1b_B1.1*** | *Morogoro region, Mara region* | *2010* | *2013* | *0.07* | *0.02* | *7* |
| ***Cosmopolitan AF1b_B1.1.1*** | *NA* | *2017* | *2018* | *0.07* | *0.02* | *3* |
| ***Cosmopolitan AF1b_B1.2*** | *Mara region* | *2010* | *2013* | *0.07* | *0.02* | *21* |
| ***Cosmopolitan AF1b_B1.3*** | *Mara region* | *2011* | *2017* | *0.07* | *0.02* | *28* |
| ***Cosmopolitan AF1b_C1*** | *Mara region* | *2012* | *2013* | *0.07* | *0.02* | *2* |
| ***Cosmopolitan AF1b_C1.1*** | *Mara region* | *2011* | *2012* | *0.07* | *0.02* | *5* |
| ***Cosmopolitan AF1b_C1.1.1*** | *Morogoro region, Mara region* | *2010* | *2013* | *0.07* | *0.02* | *35* |
| ***Cosmopolitan AF1b_D1*** | *Dar es Salaam, Morogoro region, Mara region* | *2010* | *2012* | *0.07* | *0.02* | *14* |
| ***Cosmopolitan AF1b_E1*** | *Mara region* | *2010* | *2013* | *0.07* | *0.02* | *23* |
