# Supplementary material for: Making genomic surveillance deliver: A lineage classification and nomenclature system to inform rabies elimination
Source: PLoS Pathog. 2022 May 2;18(5):e1010023. doi: 10.1371/journal.ppat.1010023 (PMC9162366; doi:10.1371/journal.ppat.1010023)
Supplement: S3 Table — Details of the 15 global MAD DOG lineages that have been detected in Tanzania. Includes all places sequences assigned to each lineage have been seen in, the first and most recent collection years of those sequences and the number of sequences assigned to each lineage. (DOCX) [file ppat.1010023.s003.docx]

| ***lineage*** | ***place*** | ***year_first*** | ***year_last*** | ***n_seqs*** |
| --- | --- | --- | --- | --- |
| ***A1.2*** | *Chake chake, Kusini Unguja)* | *2010* | *2017* | *3* |
| ***A1.2.1*** | *Dar es Salaam, Iringa region, Lindi region* | *2010* | *2011* | *4* |
| ***A1.2.2*** | *Arusha Region, Bariadi, Dar es Salaam, Iringa region, Loliondo, Loliondo Game Control Area, Morogoro region, Musoma, Ngorongoro, Mara region* | *1996* | *2017* | *46* |
| ***AF1b_A1*** | *Morogoro region, Ngorongoro, Pemba island, Serengeti, Serengeti National Park, Tarangire, Tarime* | *1994* | *2017* | *13* |
| ***AF1b_A1.1*** | *Mara region* | *2011* | *2013* | *2* |
| ***AF1b_A1.1.1*** | *Pwani region, Mara region* | *2008* | *2018* | *11* |
| ***AF1b_A1.1.2*** | *Arusha Region, Morogoro region, Mtwara region, Pemba island, Mara region* | *2003* | *2011* | *6* |
| ***AF1b_B1*** | *Mara region* | *2004* | *2012* | *3* |
| ***AF1b_B1.1*** | *Morogoro region, Mara region* | *2010* | *2018* | *10* |
| ***AF1b_B1.1.1*** | *Morogoro region, Mara region* | *2010* | *2013* | *42* |
| ***AF1b_B1.2*** | *Mara region* | *2011* | *2017* | *25* |
| ***AF1b_B1.3*** | *Mara region* | *2010* | *2013* | *21* |
| ***AF1b_C1*** | *Lindi region, Pemba island, Mara region* | *2009* | *2017* | *10* |
| ***G1*** | *Mara region* | *2010* | *2013* | *24* |
| ***K1*** | *Serengeti, Serengeti National Park* | *1997* | *1999* | *4* |
