# Supplementary material for: Making genomic surveillance deliver: A lineage classification and nomenclature system to inform rabies elimination
Source: PLoS Pathog. 2022 May 2;18(5):e1010023. doi: 10.1371/journal.ppat.1010023 (PMC9162366; doi:10.1371/journal.ppat.1010023)
Supplement: S4 Table — Details of potentially emerging or undersampled lineages in the Cosmopolitan clade that do not yet have enough sequences to be defined as a new lineage. Lineages are named with an ‘_Ex’ suffix (where x is a number indicating multiple distinct emerging/undersampled lineages within the same parent lineage). The number of tips for each lineage, as well as the country and time period the sequences are from are also listed, as is the patristic distance between the node defining the lineage and its parent node. (DOCX) [file ppat.1010023.s004.docx]

| ***lineage*** | ***tips*** | ***patristic_distance*** | ***country*** | ***year_first*** | ***year_last*** |
| --- | --- | --- | --- | --- | --- |
| ***Cosmopolitan AM1_A1_E1*** | ***5*** | ***0.0787338298*** | ***United States*** | ***2009*** | ***2009*** |
| ***Cosmopolitan AM3a_A1_E1*** | ***7*** | ***0.0897059767*** | ***Brazil*** | ***2006*** | ***2006*** |
| ***Cosmopolitan AM3a_A1_E2*** | ***5*** | ***0.0903899673*** | ***Brazil*** | ***2006*** | ***2009*** |
| ***Cosmopolitan AM3a_A1_E3*** | ***6*** | ***0.092716402*** | ***Brazil*** | ***2006*** | ***2009*** |
| ***Cosmopolitan AM3a_A1.1_E1*** | ***6*** | ***0.0992595827*** | ***Brazil*** | ***2007*** | ***2007*** |
| ***Cosmopolitan AM3b_A1_E1*** | ***7*** | ***0.1052622533*** | ***Brazil*** | ***2006*** | ***2009*** |
| ***Cosmopolitan AM3b_A1.1.1_E1*** | ***7*** | ***0.1021828483*** | ***Brazil*** | ***2006*** | ***2009*** |
| ***Cosmopolitan CA1_A1_E1*** | ***7*** | ***0.0651955575*** | ***Russia*** | ***2012*** | ***2014*** |
| ***Cosmopolitan CA1_A1_E2*** | ***6*** | ***0.0694951132*** | ***China*** | ***2014*** | ***2018*** |
| ***Cosmopolitan CA1_A1.1.1_E1*** | ***5*** | ***0.0697324895999999*** | ***Mongolia*** | ***2005*** | ***2008*** |
| ***Cosmopolitan CA1_B1.1_E1*** | ***7*** | ***0.0749520980999999*** | ***Mongolia*** | ***2017*** | ***2018*** |
| ***Cosmopolitan CA1_B1.1_E2*** | ***9*** | ***0.0779361549999999*** | ***China*** | ***2014*** | ***2014*** |
| ***Cosmopolitan CA2_A1_E1*** | ***6*** | ***0.054733071*** | ***Georgia*** | ***2015*** | ***2015*** |
| ***Cosmopolitan CA2_A1_E2*** | ***6*** | ***0.0525030796*** | ***Georgia*** | ***2015*** | ***2015*** |
| ***Cosmopolitan CA2_A1_E3*** | ***8*** | ***0.0521217738*** | ***Georgia*** | ***2015*** | ***2016*** |
| ***Cosmopolitan EE_A1_E1*** | ***5*** | ***0.0594515072*** | ***Serbia*** | ***1997*** | ***1997*** |
| ***Cosmopolitan EE_A1_E2*** | ***6*** | ***0.0722742704*** | ***Serbia*** | ***1997*** | ***2000*** |
| ***Cosmopolitan EE_A1_E3*** | ***7*** | ***0.0715864755*** | ***Serbia*** | ***1998*** | ***2000*** |
| ***Cosmopolitan_A1.2.1_E1*** | ***6*** | ***0.0495317759*** | ***South Africa*** | ***2009*** | ***2009*** |
| ***Cosmopolitan_A1.2.2_E1*** | ***5*** | ***0.0648515310999999*** | ***Tanzania*** | ***2003*** | ***2004*** |
| ***Cosmopolitan_B1.1_E1*** | ***9*** | ***0.0614716492*** | ***Iran*** | ***2013*** | ***2013*** |
| ***Cosmopolitan_B1.1_E2*** | ***7*** | ***0.0610973553*** | ***France*** | ***1991*** | ***1991*** |
| ***Cosmopolitan_F1.1_E1*** | ***7*** | ***0.0593858836999999*** | ***Israel*** | ***1996*** | ***1998*** |
| ***Cosmopolitan_B1.1.1_E1*** | ***5*** | ***0.0540722183*** | ***Cameroon*** | ***2009*** | ***2011*** |
