# Supplementary material for: Making genomic surveillance deliver: A lineage classification and nomenclature system to inform rabies elimination
Source: PLoS Pathog. 2022 May 2;18(5):e1010023. doi: 10.1371/journal.ppat.1010023 (PMC9162366; doi:10.1371/journal.ppat.1010023)
Supplement: S5 Table — Details of singleton sequences with long branch lengths (longest 5%), indicating potentially undersampled lineages, sequencing errors, or newly emerging divergent lineages that need to be monitored. The number of singleton sequences in each lineage is listed, plus collection years and counties of those singleton sequences where this information is publicly available. (DOCX) [file ppat.1010023.s005.docx]

| ***lineage*** | ***n_singletons*** | ***singleton_countries*** | ***singleton_years*** |
| --- | --- | --- | --- |
| ***Cosmopolitan AF1a_A1*** | ***1*** | ***-*** | ***1995*** |
| ***Cosmopolitan AF1b_A1*** | ***2*** | ***-, Uganda*** | ***1995, 2011*** |
| ***Cosmopolitan AM1_A1*** | ***1*** | ***-*** | ***2016*** |
| ***Cosmopolitan AM3a_A1*** | ***2*** | ***Brazil*** | ***2006, 2007*** |
| ***Cosmopolitan AM3b_A1*** | ***2*** | ***Brazil*** | ***2006*** |
| ***Cosmopolitan AM3b_A1.1.1*** | ***4*** | ***Brazil*** | ***2006*** |
| ***Cosmopolitan AM4_A1*** | ***1*** | ***United States*** | ***1997*** |
| ***Cosmopolitan CA1_A1*** | ***2*** | ***Kazakhstan, -*** | ***2004, 2014*** |
| ***Cosmopolitan CA2_A1*** | ***1*** | ***Iran*** | ***2005*** |
| ***Cosmopolitan_A1.1.2*** | ***1*** | ***Iran*** | ***2005*** |
| ***Cosmopolitan_A1.2.3*** | ***2*** | ***-*** | ***1995*** |
| ***Cosmopolitan_A1.3.1*** | ***1*** | ***-*** | ***2016*** |
| ***Cosmopolitan_B1.1*** | ***1*** | ***-*** | ***1999*** |
| ***Cosmopolitan_E1*** | ***1*** | ***Iran*** | ***2005*** |
| ***Cosmopolitan_H1*** | ***2*** | ***Costa Rica, -*** | ***2014, 2016*** |
| ***Cosmopolitan_A1.3.2*** | ***1*** | ***Brazil*** | ***2007*** |
