# Supplementary figures and images for: Making genomic surveillance deliver: A lineage classification and nomenclature system to inform rabies elimination
Source: PLoS Pathog. 2022 May 2;18(5):e1010023. doi: 10.1371/journal.ppat.1010023 (PMC9162366; doi:10.1371/journal.ppat.1010023)

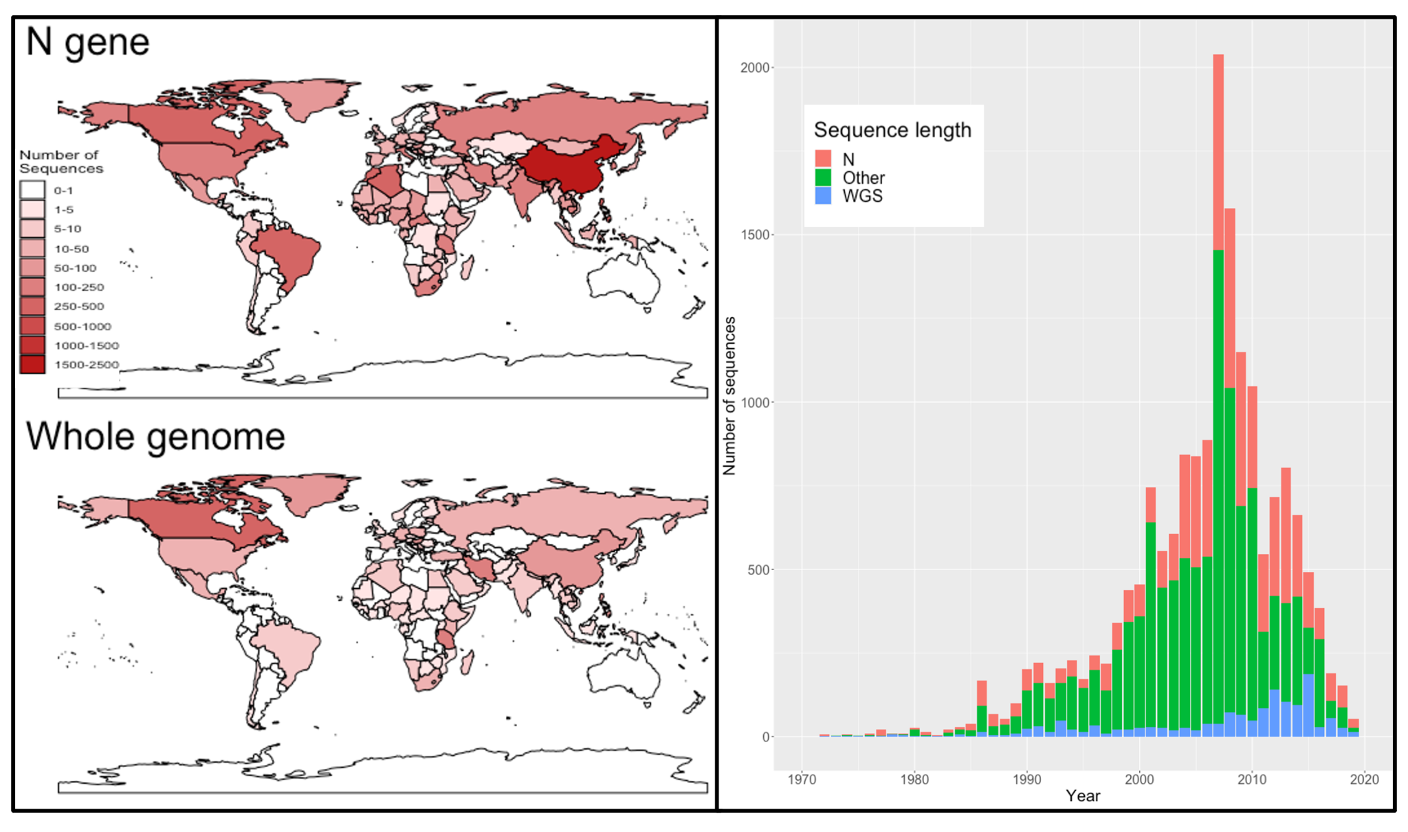

Supplement: S1 Fig — Left: Map of collection locations of all available whole genome and N gene sequences. Base layer of map from Natural Earth (https://www.naturalearthdata.com/). Right: Time series of number of whole genome, N gene, and shorter sequences collected. (TIF) [file ppat.1010023.s006.tif]

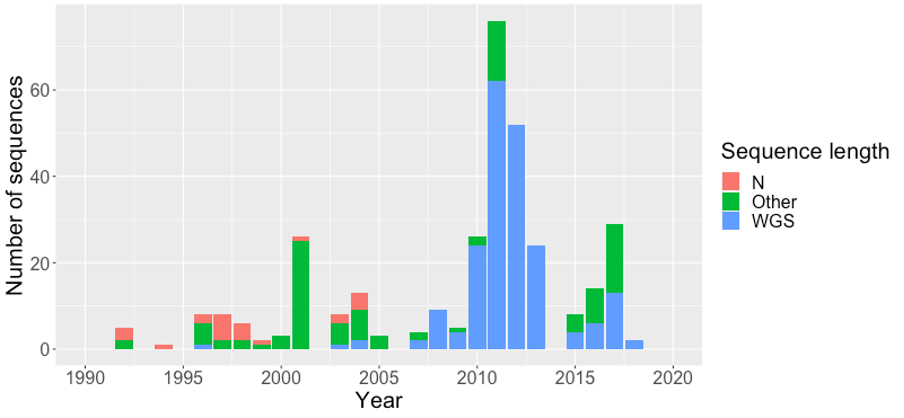

Supplement: S2 Fig — Timeseries of the number of whole genome, N gene, and shorter partial genome sequences from Tanzania available on RABV-GLUE. (TIF) [file ppat.1010023.s007.tif]
