# Supplementary material for: Making genomic surveillance deliver: A lineage classification and nomenclature system to inform rabies elimination
Source: PLoS Pathog. 2022 May 2;18(5):e1010023. doi: 10.1371/journal.ppat.1010023 (PMC9162366; doi:10.1371/journal.ppat.1010023)
Supplement: Attachment — Submitted filename: Review responses (1).docx [file ppat.1010023.s008.docx]

**Cover Letter**

Manuscript ID: PPATHOGENS-D-21-02065

**Re: “Making Genomic Surveillance Deliver: a Lineage Classification and Nomenclature System to Inform Rabies Elimination”**

Dear Editor,

We thank the reviewers for their constructive feedback. We have detailed our point-by-point responses to each of the reviewers comments below.

During the review process and in response to valuable insights from the reviewers we further identified two major improvements to the designation process: 1) to better integrate WGS and N gene data which and 2) to collapse the ladder-like structure of consecutive lineage iterations with only one sequence difference (following useful comments from reviewer 3), instead requiring that each new lineage have at least 10 sequences. As a result the number and names of lineages have now changed, and as such some of the comments raised by the reviewers have now become redundant. We explain this in relation to specific reviewer comments in the point-by-point response below. Values within the text, as well as figures and tables, have been updated to reflect the changes to the names and numbers of lineage as a result of the updated designation process.

An additional set of outputs is now also generated and discussed, indicating emerging/undersampled lineages and singletons of interest. This addresses multiple reviewer comments and is further detailed in the responses below.

We hope you find our responses to the reviewers comments agreeable and that our manuscript is acceptable for publication. We thank the reviewers again for their valuable time and feedback.

Yours faithfully,

Kathryn Campbell,

University of Glasgow,

On behalf of co-authors

**Part I - Summary**

**Reviewer #1:** The manuscript „Making Genomic Surveillance Deliver: A Lineage Classification and Nomenclature System to Inform Rabies Elimination“ by Campbell and coauthors describes a high resolution phylogenetic classification system for rabies to identify incursions and transmission routes. The proposed model is based on the experiences with SARS-CoV-2 and could also be used for other epizootics/enzootics. Optimization of the classification of outbreak strains is important and improvements seem helpful. A major strength of the presented workflow is the inclusion of WGS and partial sequences (like the N gene) as well as a more structured and more detailed classification which could help to compare genetic epidemiology analysis of different working groups in the future.

However, it remains unclear, what the advantages for the genomic epidemiology and control measures are. Statements about the need of improved classifications should be more clear and more detailed. Today, analysis of transmission routes is done for rabies in numerous studies (e.g. Denise A Marston, Daniel L Horton, Javier Nunez, Richard J Ellis, Richard J Orton, Nicholas Johnson, Ashley C Banyard, Lorraine M McElhinney, Conrad M Freuling, Müge Fırat, Nil Ünal, Thomas Müller, Xavier de Lamballerie, Anthony R Fooks, Genetic analysis of a rabies virus host shift event reveals within-host viral dynamics in a new host, Virus Evolution, Volume 3, Issue 2, July 2017, vex038, <https://doi.org/10.1093/ve/vex038>). In those studies, phylogenetic or BEAST analyses allowed highly detailed data and conclusions.

In the manuscript, it must therefore be made even clearer what the advantages of the new methodology are. Overall, the structure of the manuscript takes some getting used to and should be reconsidered.

*Thank you for these valuable comments. In revising the manuscript we have further elaborated on how WGS and N gene data can be effectively combined using our proposed lineage designation and assignment tool, and we have further made clear how the tool is not meant to replace detailed phylodynamic studies. In our revision we hope the structure is now clearer, taking on board the feedback from all three reviewers.*

**Reviewer #2:** Campbell et al. develop a system to classify rabies virus sequences into lineages, adapting the existing PANGO lineage method developed for SARS-CoV-2. The authors describe this new method and provide example cases that highlight the utility of more fine-grained classifications for rabies surveillance and transmission analysis. Overall, the paper is clearly written, the classification system makes sense, and the examples provided are a nice addition that really highlights the method concretely. Additionally, the comparison between resolution from WGS compared to N gene is helpful. I have only minor comments.

*Thank you - we really appreciate this feedback.*

**Reviewer #3:** In this work, Campbell et al. applied a dynamic lineage classification and nomenclature system previously used for SARS-CoV-2 on rabies virus (RABV) sequence data, specifically for the Cosmopolitan clade of viruses. Compared to the present system which only provides a high-level subspecies classification, the finer delineations produced by the authors facilitate epidemiological interpretations such as origins identification and surveillance of global as well as local circulating strains. The manuscript is generally well-written with a clear presentation on how the newly applied system could be used to partially describe the spatiotemporal dynamics of RABV.

*Thank you.*

**Part II – Major Issues: Key Experiments Required for Acceptance**

**Reviewer #1:** 1. Please explain the term „…we find an over 200% increase in the definition…“. What does the 200% refer to (- Line 57 and 318)? Is this changing the resolution of the analysis possible today or does this just change and unify the classifications?

*As indicated in the cover letter, we managed to update the way the lineage designation occurs so that N gene designations are no longer separately classified; instead these add additional lineages onto the whole genome designations. Additionally, our change to the lineage designation rules collapses the ladder-like structure of multiple lineage iterations with only one sequence difference i.e. reducing artificial definition. These details are clarified in the methods. However, this results in a change in the total number of lineages and hence this statement no longer applies. More generally we have revised the detail on the resolution of analyses to be clearer within the author summary and discussion (the lines indicated in the comment).*

2. How was it proven, that there were areas of persistence and transmission that were not previously apparent? Please provide data and explanations (- Line 58)

*We have revised this sentence to refer instead to: areas of persistence and routes of virus movement, including transboundary incursions. The areas of persistence refer to the lineages sustained through time when examining our example from Serengeti district in Tanzania, whilst our cases studies provide examples of possible routes of virus movement (both in Africa and the Americas).*

3. Would a „standard analysis“ using Blast, MEGA and BEAST result in the same outcome (as e.g. in Figure 6)? Please provide a direct comparison to convince the reader to use the new classification tool. What is the advantage of the strain designation for this type of analysis of a standard tree and BEAST analysis (e.g. Figure 7)? Please provide more detailed explanations/data of the advantages.

*The maps of the case studies in figure 6 are not temporal, or in fact phylogenetic, analyses, although the relevant subsection of the phylogeny is shown for reference. They instead identify the first year a particular lineage was isolated from the countries of interest. Therefore, other phylogenetic applications would pull out similar information, but not in the systematic, objective way that MAD DOG does. Additionally, the manual curation of RABV-GLUE facilitates the data curation used for these analyses. The phylogenetic component is important for the initial lineage designation. Although a BEAST analysis may produce more robust, temporally scaled trees, the analysis simply takes too long to be appropriate for the aims of MAD DOG. The focus of the tool is to provide a rapid result with an overview of viral diversity, which can be used to provide insight to direct further analyses, such as in BEAST, or to provide a rapid result to be used for actionable information in the event of identifying incursions and informing control policies. This is similar to, for example, methods employed by NextStrain to provide a level of phylogenetic inference not limited by time constraints. This has now been detailed in the discussion section to highlight the limitations of a standard phylogenetic approach versus a BEAST analysis, and why it is more appropriate in this case.*

4. It is not clear why the proposed new classification system is supporting the „Zero by 30“ aim. Is this view supported by WHO/FAO and rabies reference laboratories?

*MADDOG aims to provide a systematic classification system that can inform control programmes by facilitating genomic analysis in a way that can be easily applied by public and vet health researchers. We hope that this classification system will be useful by revealing incursions and persistent transmission, which have relevance to rabies control programmes. However, we have now removed this clause as we do not mean to imply that there is official tripartite support for our proposed methods.*

**Reviewer #3:** However, there are a couple of concerns I hope the authors will find helpful in improving their work:

1) I am presuming that the phylogenetic trees shown in Figure 4 are patristic distance trees and not temporally-resolved ones. While there isn’t a scale to denote the branch lengths, a fair proportion of tips appear as singletons with extended branch lengths from the common ancestor of the virus clade they have been assigned to. These tips likely represent undersampled diversity. While these singletons may share common mutations with the larger clades that they are assigned to, they should also encode multiple mutations that would otherwise be deemed genetically distinct should there be more similar sequences sampled. Such singletons are less likely to appear for SARS-CoV-2 given its recent emergence and the unprecedented volume of sequence data that have been generated in substantially shorter timescales. As such, I feel that there is a need to tweak the current lineage classification system to flag and distinguish these singletons, which in itself may help inform on where there might be surveillance gaps.

*Thank you, this was an excellent insight. We have now added a scale to all the main phylogenies inferred for this work. We have also included an ‘Emerging or Unsampled Lineages’ output that identifies divergent clusters of 5-9 sequences that may not be designated as lineages due to undersampling resulting in too few sequences, or may be emerging and will be designated in time. The output lists the current lineage name followed by Ex where x is a number indicating separate emerging/undersampled lineages within the same lineage. Additionally, we include a ‘Singletons of Interest’ output, which identifies singletons and lists them as lineages that need to be monitored or researched as they may be emerging, undersampled or contain sequencing errors. (Table S4 and S5). This is now detailed in the methods and explored in the results and discussion.*

Although the authors did not address this specifically, they note that the sparse availability of RABV sequences meant that the new nomenclature “do not fully reflect the depth of diversity and evolutionary time frame” of the viruses. The authors then made the argument that this limitation would only affect the naming of lineages, and that “analyses should be designed to incorporate the iterative nature of lineage designations”. However, if the aim of this proposed system is to be a new standard to capture underlying evolutionary and/or epidemiological processes which the authors assert, more care should be taken into the precision of what evolutionary/epidemiological bounds are embedded within these names.

*Agreed. We have tried to emphasise the importance of following the full evolutionary history of lineages when drawing comparisons. To assist this, we have updated the ‘lineage info’ table output so that it now includes an ‘evolution’ column that details the iterations leading to each lineage. E.g. (AF1a_A1.2<-AF1a_A1<-A1<-Cosmopolitan).*

2) I am also curious as to the choice of outgroup used to root the tree. Is the tree currently rooted by temporal structure? If so, how robust is the temporal structure? If the authors claim that the new nomenclature system can help identify case origins and reconstruct historical spread, the rooting choice isn’t a trivial one as it will impact interpretations on the directionality of evolutionary events and reconstruction of ancestral states.

*The tree is not timescaled and so is not rooted by temporal structure. The outgroup is instead 10 sequences from a different clade (Asian SEA1b) to ensure the structure of the Cosmopolitan subclades are correct in relation to each other. We have now included a scale on the tree in figure 4 indicating substitutions per site per year. We would consider investigating the results from a temporally structured tree in future work, however as RABV is endemic, this is more complicated than for SARS-CoV-2.*

**Part III – Minor Issues: Editorial and Data Presentation Modifications**

**Reviewer #1:** 1. Box 1: what is the difference/relation of clade and lineage should be explained. Please define also clade designation and clade assignment.

*We have clarified the relationship between clades and lineages in the definitions box, specifying that clades and subclades can be split further into lineages, which are the high resolution classification.*

*We have also now explained that clades and subclades are already defined for rabies virus, whereas lineages are not and thus is where we begin the classification process in this study. We use clade designations provided by Troupin et al. (2016) (see the tree in Fig 1 in box 1) which are widely used in the literature. These clade designations are recapitulated when MAD DOG is run, and then further built upon to produce the lineage designations.*

*Clade designation and assignment are not included here as we use the Troupin et al. classifications, which are widely used in the literature. Designation refers to classifying a set of sequences based on a set of rules and naming them accordingly. There is no specific set of rules for defining a rabies virus clade, hence why this new system is so important.*

2. Figure 3/step 3: how to deal with new outbreaks with novel lineages and less than 10 sequences in the early phase?

*At present, any lineages with less than 10 sequences are contained within their parent lineage. We have now included an ‘Emerging or Unsampled Lineages’ output that identifies divergent clusters of 5-9 sequences that may not be designated as lineages due to undersampling resulting in too few sequences, or may be emerging and will be designated in time. The output lists the current lineage name followed by Ex where x is a number indicating separate emerging/undersampled lineages within the same lineage. Additionally, we include a ‘Singletons of Interest’ output, which identifies singletons and lists them as lineages that need to be monitored or researched as they may be emerging, undersampled or contain sequencing errors. (Table S4 and S5). This is now detailed in the methods and explored in the results and discussion.*

3. The keywords listed should be checked. Words like phylogenetics or lineage are missing.

*Great suggestion - phylogenetics has been added to the keywords list. Lineage and other relevant terms are included in the title, so we chose not to include these as keywords too.*

4. The boxes seem to be missplaced in the introduction section.

*The paper contains some complex and new terminology throughout, which we feel benefits the reader to be introduced to early on to make the subsequent sections clearer. We have therefore now pointed to box 1 early in our introduction. However, Box 2 was in the wrong place and we have moved it down to after RABV-GLUE is first mentioned in the methods. RABV-GLUE is essential to understand the data acquisition process in the methods, and hopefully this change helps make the flow clearer.*

5. Figure 3 seems to be more part of the results section

*The methods for this study are quite complex and we therefore included figure 3 to summarise the text in the methods section with the aim of making this section easier to follow.*

6. Figure 3/step 4: Single nucleotide changes are used for classification. How to exclude sequencing errors as a reason for new „lineages“? How robust is the analysis concerning sequencing errors?

*The requirement of the lineage designation to have at least 10 sequences that all share a nucleotide change from their ancestor limits artificial lineages due to sequencing errors. As it is unlikely that at least 10 sequences would have the same error, we feel the system is therefore robust against this. We have now indicated in the methods why we chose a minimum of 10 sequences.*

7. Define „cosmopolitan clade“

*Great point - this is now added to the definitions box.*

8. Several of the supplemental datasets (figures/tabes) are not mentioned within the text.

*The supplementary materials have been edited in line with the updated classifications mentioned in the cover letter and the inclusion of emerging/undersampled lineages and singletons of interest. These have now all been referred to within the main text.*

**Reviewer #2:** 1. The authors mention in the Methods that MADDOG is available via an R package, which is great. However, the authors do not provide a link to the code, installation instructions, an example dataset, or any sort of documentation. Because this paper primarily introduces a new method, it would be helpful if the authors provided a direct link to the method, even if it is just a github page. I was not able to find such a page from a google search, and I think it would be helpful to potential users to provide at minimum a link to a web page with some documentation, an example dataset, and installation instructions.

*A DOI to the Zenodo release is included in the MAD DOG part of the methods section (DOI:* [*10.5281/zenodo.5503916*](https://doi.org/10.5281/zenodo.5503916)*). Complete installation and usage instructions are available in the readme file as well as how to use the example dataset included in the tool. The link to the corresponding GitHub repository is also available on the zenodo page.*

2. Figure 5 is a bit challenging to interpret as currently colored and described in the legend. From reading the legend, I had inferred that when a given country had 2 circles overlaid on its centroid, that the darker/more vibrant one represented lineages inferred from WGS, while the lighter one represented those inferred from N gene sequences. However, some countries have multiple overlaid circles that are all light (Brazil, US, Mexico). For these countries, it was not clear to me what the interior vs. exterior circles represented. Additionally, the colors in the less vibrant circles were challenging to distinguish. I would suggest clarifying this in the legend, and perhaps altering the figure in some way to make the colors easier to distinguish for the light circles.

*We agree! We had been struggling to find the best way to present this much data in one figure. Now the N gene designations have been updated to correspond to the WGS designations, this all fits much better on the one map without the need for double circles or overlays.*

3. The case examples detailed in figures 6 and 7 were excellent and very helpful. However, showing some additional data would be helpful in showing how the sequence data and lineage designations allow for the inferences shown in the map and arrow graphics. For figure 6, it would be helpful to include the tree or at least a partial snippet of the tree, so that the data used for the inference shown on the map is clear. For figure 7b, it would be helpful to show on country-level information for the tips shown on the tree. This could be added as additional text in the strain name, as shapes or colors for the tips, or some other method. Having this information directly on the tree would make it more clear how the tree was used to infer the transmission history shown in 7a.

*Figure 7 has been updated to include the subtrees, with icons indicating country of origin and dated tips for each sequence as well as a readable scale bar showing substitutions per site per year. As detailed in the cover letter, due to changes in the addition of N gene data and collapsing ladder like structures, the names of lineages in figure 7 are now different, but these still represent the same lineages.*

This is just a compliment: I thought it was very cool that the authors followed up with the original authors of the case report described in example 1 to confirm their inferences! That was a fun example to read.

*Thank you! We’re hoping this new system will allow us to identify other instances like this! Incidentally, RABV-GLUE contains key metadata which can be updated and changes recorded when such errors are found like this.*

**Reviewer #3:** Lastly, a minor point - can the authors provide a main or supplementary figure showing the difference between the current and proposed lineage designation? I think it will be impactful to show how the new nomenclature system yield more informative delineations compared to the currently used one.

*Thank you for this excellent suggestion! This has now been included within figure 4, indicating the increased definition from the old classifications to the new lineage designations at whole genome level, and further showing the added definition provided by including N gene sequences.*
